# Accelerating the in vitro emulation of Alzheimer’s disease-associated phenotypes using a novel 3D blood-brain barrier neurosphere co-culture model

**DOI:** 10.3389/fbioe.2023.1251195

**Published:** 2023-10-09

**Authors:** Eunkyung Clare Ko, Sarah Spitz, Francesca Michela Pramotton, Olivia M. Barr, Ciana Xu, Georgios Pavlou, Shun Zhang, Alice Tsai, Anna Maaser-Hecker, Mehdi Jorfi, Se Hoon Choi, Rudolph E. Tanzi, Roger D. Kamm

**Affiliations:** ^1^ Department of Mechanical Engineering and Biological Engineering, Massachusetts Institute of Technology, Cambridge, MA, United States; ^2^ Genetics and Aging Research Unit, McCance Center for Brain Health, Mass General Institute for Neurodegenerative Disease, Department of Neurology, Massachusetts General Hospital and Harvard Medical School, Charlestown, MA, United States

**Keywords:** Alzheimer’s disease (AD), blood-brain barrier (BBB), neurospheres, microfluidics, microphysiological system (MPS)

## Abstract

High failure rates in clinical trials for neurodegenerative disorders such as Alzheimer’s disease have been linked to an insufficient predictive validity of current animal-based disease models. This has created an increasing demand for alternative, human-based models capable of emulating key pathological phenotypes *in vitro*. Here, a three-dimensional Alzheimer’s disease model was developed using a compartmentalized microfluidic device that combines a self-assembled microvascular network of the human blood-brain barrier with neurospheres derived from Alzheimer’s disease-specific neural progenitor cells. To shorten microfluidic co-culture times, neurospheres were pre-differentiated for 21 days to express Alzheimer’s disease-specific pathological phenotypes prior to the introduction into the microfluidic device. In agreement with post-mortem studies and Alzheimer’s disease *in vivo* models, after 7 days of co-culture with pre-differentiated Alzheimer’s disease-specific neurospheres, the three-dimensional blood-brain barrier network exhibited significant changes in barrier permeability and morphology. Furthermore, vascular networks in co-culture with Alzheimer’s disease-specific microtissues displayed localized β-amyloid deposition. Thus, by interconnecting a microvascular network of the blood-brain barrier with pre-differentiated neurospheres the presented model holds immense potential for replicating key neurovascular phenotypes of neurodegenerative disorders *in vitro*.

## Introduction

Alzheimer’s disease (AD) constitutes the most common neurodegenerative disorder, affecting around 24 million people worldwide. In addition to the key neuropathological hallmarks of AD including amyloid plaques, neurofibrillary tangles, glial activation, and neuronal loss, early blood-brain barrier (BBB) breakdown and vascular dysregulation have been reported *in vivo* (Serrano-Pozo et al., 2011; Zenaro et al., 2017). Comprised of microvascular endothelial cells, brain pericytes, and astrocytes the BBB is a highly selective barrier that mediates the transport of molecules (e.g., therapeutic compounds) between the bloodstream and the brain interstitium. Consequently, vascular dysregulation can have detrimental effects on brain tissue homeostasis and thus disease onset and progression, rendering the BBB a key player in AD (Sweeney et al., 2018). While animal-based *in vivo* models have yielded valuable insights into AD pathology, the models’ low predictive validity has restricted the success rate of clinical trials (Chin, 2011; Boutajangout and Wisniewski, 2014; Dujardin et al., 2015; Puzzo et al., 2015; Drummond and Wisniewski, 2017). To address this issue the Food and Drug Administration (FDA) has recently approved the use of cell-based assays and computer models in pre-clinical trials by passing the FDA Modernization Act 2.0. To that end, specific focus has been directed toward the development of human cell-based *in vitro* models in recent years. While early models combined Transwell^®^ culture set-ups with the external addition of amyloid β (Aβ)-peptides (Qosa et al., 2014), recent advances in biological engineering and organ-on-a-chip technology have allowed for the creation of intricate co-culture models that better mimic (patho-)physiological tissue niches *in vitro* (Park et al., 2015; Shin et al., 2019). For example, we have previously shown that neurospheres derived from AD-specific neural progenitor cells carrying familial AD mutations in amyloid precursor protein (APP) and presenilin 1 (PSEN1) replicate disease-specific phenotypes *in vitro* including increased secretion of Aβ42-peptides and elevated Aβ42/40 ratios (Jorfi et al., 2018). Furthermore, we have developed a perfusable microvascular network model of the human BBB by co-cultivating primary human microvascular endothelial cells, brain pericytes, and astrocytes in a fibrin hydrogel matrix (Campisi et al., 2018; Hajal et al., 2022).

**FIGURE 1:**
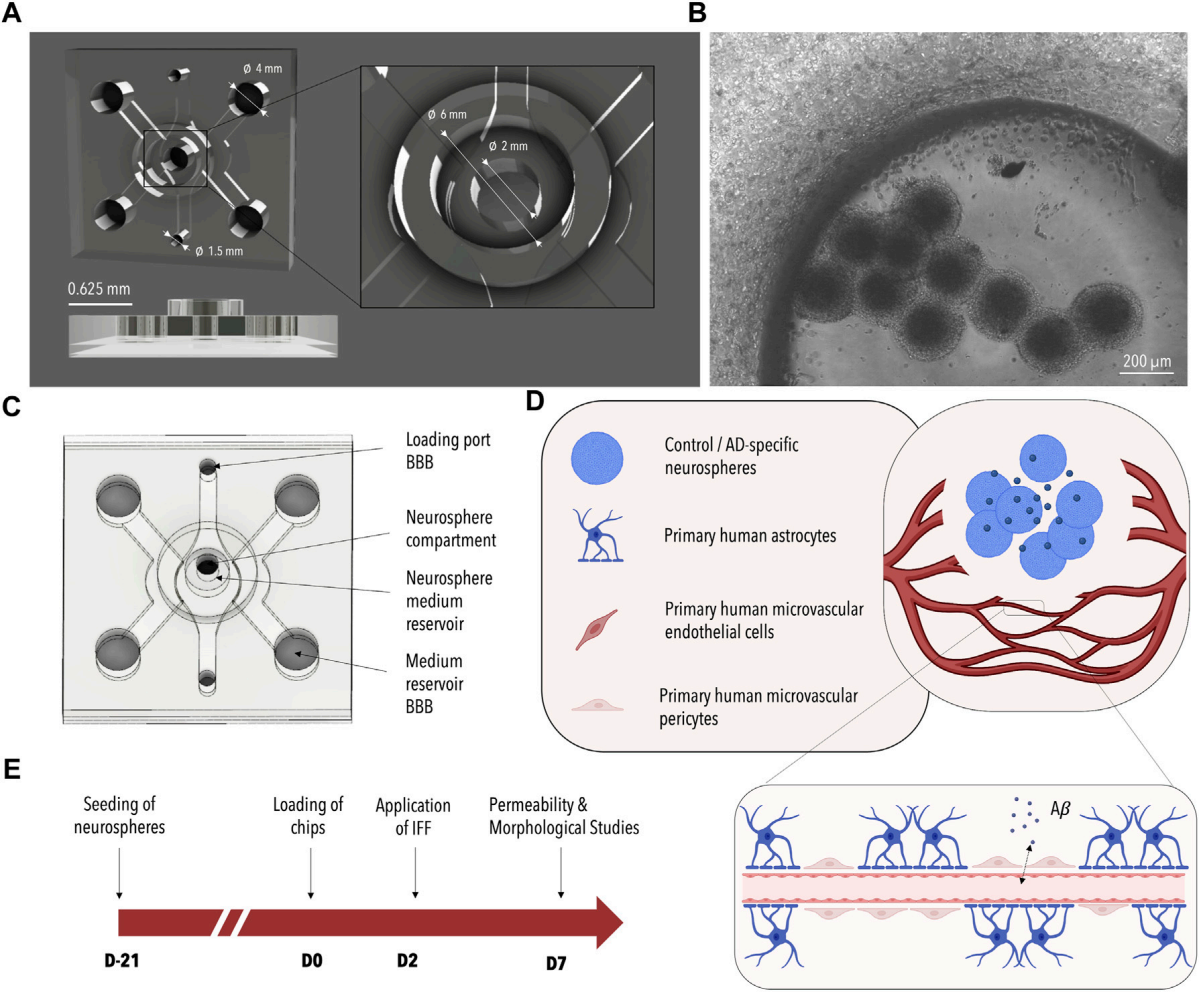
Design of the 3D-AD Microfluidic Device and Experimental Setup. **(A)** 3D rendered image of the microfluidic device consisting of a main BBB chamber with two side channels and an additional neurosphere compartment located in the center of the BBB chamber. Dimensions are indicated in white. **(B)** Phase contrast image of the BBB cells and neurospheres seeded in the device (Figure 3A bottom). **(C)** Schematic illustration of the microfluidic device. The suspended cells are injected through the BBB loading port; BBB media is added to the two media channels flanking the central chamber. The neurospheres are seeded into the central neurosphere compartment and the neuronal medium is added from the top. **(D)** Illustration of the co-culture system. ReN-AD neurospheres seeded in the central compartment secrete factors (e.g., Aβ) that induce pathological changes in the BBB microvascular network formed with primary human microvascular endothelial cells, primary human astrocytes, and primary human microvascular pericytes. **(E)** Timeline of the experiment: neurospheres are generated 21 days before initiating co-culture with the BBB on day 0. A pressure gradient is initiated on day 2 and the system is analyzed on day 7. Created with BioRender.com.

One of the drawbacks in the development of disease models, however, is that the generation of disease-specific phenotypes using neuronal microtissues (e.g., organoids) requires several weeks of culture (Choi et al., 2014; Kim et al., 2015; Jorfi et al., 2018). Aside from long culture times, different cell requirements among the individual constituents of the neurovascular unit including hydrogel matrices and cell culture media have hindered their successful co-culture. To address these issues, we previously employed a sequential seeding approach, coupled with physically separated and parallelly aligned cultivation channels to observe Aβ-mediated changes within an endothelial monolayer (Shin et al., 2019). Recognizing the need for a more streamlined process that allows for direct cell-cell interactions as well as enhanced physiological mimicry, we devised a strategy to pre-differentiate neurons in AD-specific microtissues, which subsequently could easily be injected into a microfluidic device and co-cultured with microvascular networks of the BBB once exhibiting pathological phenotypes. To accommodate these requirements, a multi-compartmentalized microfluidic platform for the co-culture of neuronal cells and BBB cells was developed as part of this study (Figures 1A–C).

We find that by introducing neuronal microtissues derived from a genetically modified neural progenitor cell line carrying two AD-specific mutations in APP and PSEN1 into the compartmentalized platform, neuron-secreted Aβ is delivered to the BBB network at sufficient concentrations to produce changes in network morphology as well as barrier permeability and results in localized Aβ deposition (Figures 1D, E). We suggest that our microphysiological model serves as a useful tool to emulate and subsequently study pathological changes within the neurovascular unit *in vitro*.

## Materials and methods

### Design and fabrication of the microfluidic co-culture platform

The microfluidic co-culture platform was designed using the computer-aided design (CAD) program AutoCAD (^©^2022 Autodesk, Inc.; T.113.M.246). Microphysiological systems were fabricated by casting polydimethylsiloxane (PDMS) (1:10 ratio) (Sylgard^®^ 184 Silicone Elastomer Kit, Down Corning) into a computer numerical control (CNC) milled polycarbonate mold using standard soft lithography techniques. After 4 h of polymerization at 80°C, the cured PDMS was removed from the CNC-milled mold and cut into 12 individual microfluidic chambers. Microfluidic inlets and outlets, medium reservoirs, and neurosphere chambers were produced using biopsy punches of varying sizes (Ø 4 mm, Ø 1.5 mm, Ø 2 mm, Kai Medical). Cylindrical medium reservoirs for the central neurosphere compartment were generated from separately casted PDMS layers (3 mm high) using both Ø 9 mm and Ø 6 mm biopsy punches. Subsequently, cleaned (adhesive tape (Scotch)) and autoclaved microstructured PDMS top layers were bonded to cleaned glass substrates (VWR, 48366-089) using air plasma (Harrick Plasma, High Power, 2 min). Glass substrates were cleaned by submerging them into absolute ethanol and treating them in an ultrasonic bath for 10 min. Substrates were dried using pressurized air. Bonded devices were baked at 80°C overnight before usage.

### BBB cell culture

The microvascular network of the human BBB was engineered employing a tri-culture of primary human astrocytes (ScienCell, 1800), primary human brain vascular pericytes (ScienCell, 1200), and primary GFP-labeled human brain microvascular endothelial cells (Angio-Proteomie, cAP-002). Cells were expanded using their respective media: Astrocyte Medium (ScienCell, 1801), Pericyte Medium (ScienCell, 1201), VascuLife^®^ VEGF Endothelial Medium (Lifeline Cell Technology, LL-0003) supplemented with 10% fetal bovine serum (FBS) (Thermo Fisher Scientific, 26140-079). Cells were passaged using TrypLE Express (ThermoFisher, 12604021). Primary human brain vascular pericytes and primary human astrocytes were cultured on poly-L-lysine [25% v/v in phosphate-buffered saline (PBS)] (Sigma Aldrich, P4707) coated flasks; primary human brain microvascular endothelial cells were seeded onto fibronectin (30 μg/mL) (Millipore Sigma, FC010) coated surfaces. The culture media was changed every 2 days. Primary human brain vascular pericytes and primary human astrocytes were used until passage 5; primary human brain microvascular endothelial cells were employed until passage 8.

### Neurosphere generation and differentiation

ReN-Ctrl (control ReN cells expressing GFP only) and ReN-AD cells (ReN cells expressing familial AD (FAD) mutations in the APP and PSEN1 as well as GFP and mCherry) used for the formation of neurospheres were generated according to a protocol previously described by *Kim et al* (Kim et al., 2015). Before the formation of neurospheres ReN cell lines were expanded in cell culture flasks coated with Matrigel^®^ (Corning, 356230) (1:100 dilution in DMEM/F12 medium) using neural expansion medium (DMEM/F12 (Gibco, 11320-033) supplemented with 500 μL of heparin (STEMCELL Technologies, 07980), 10 mL of B27 (Gibco, 17504-044), 400 μL of bFGF (Stemgent, 03-0002), 500 μL of EGF (Sigma, E9644), and 5 mL of Penicillin-Streptomycin-Amphotericin B 100x (Lonza, 17-745E) until the cells reached 70% confluency. Three weeks before seeding, the cells were detached with TrypLE express (ThermoFisher, 12604021). After incubation at room temperature for up to 5 min, the cells were collected with prewarmed differentiation media and transferred into a 15 mL tube. After the cells were centrifuged at 1,200 rpm for 5 min, they were resuspended in ReN cell differentiation medium. The differentiation medium was generated by combining 485 mL of DMEM/F12 (Gibco, 11320-033) with 0.5 mL of heparin (2 mg/mL stock, STEMCELL Technologies, 07980), 10 mL of B27 (Gibco, 17504-044) and 5 mL of 100x penicillin/streptomycin/amphotericin B (Lonza, 17-745E). Neurospheres were generated by seeding 2 × 10^4^ cells per well in an ultra-low attachment 96-well plate (Corning^®^, 7007). During the 3 weeks of neurosphere culture, the medium was not removed but each well was supplemented with 20 μL of fresh differentiation medium every 2-3 days.

### Microfluidic co-culture

One day before initiating the co-culture in the microfluidic device, 10–12 neurospheres were collected in a microcentrifuge tube. The number of neurospheres was adjusted to match the number of cells employed in our previous microphysiological AD study (Shin et al., 2019). Equal volumes of the following three stock solutions were mixed to generate a homogenous cell suspension: 36 × 10^6^ primary human brain microvascular endothelial cells/mL, 6 × 10^6^ primary human astrocytes/mL, and 3 × 10^6^ primary human brain vascular pericytes/mL. All stock solutions were prepared using 4 U/mL of thrombin (Sigma Aldrich, T4648) in VascuLife^®^ medium (LifeLine Technologies, LL-0003) supplemented with 0.19 U/mL of heparin. 15 μL of the cell suspension were mixed with 15 µL of a 6 mg/mL fibrinogen stock solution (Sigma Aldrich, F8630) and loaded into the central BBB chamber via the BBB loading port. To prevent the hydrogel from escaping through the microfluidic device’s neurosphere chamber, a PDMS stamp was placed on top of the PDMS reservoir prior to loading. After the channel was filled, the remaining hydrogel in the neurosphere compartment was removed by aspirating 6 µL of the hydrogel mixture from the inlet of the microfluidic device. After polymerization for 15 min at 37°C and the addition of 140 µL of VascuLife^®^ medium (LifeLine Technologies, LL-0003) supplemented with 0.19 U/mL of heparin to each side of the microfluidic device, the neurosphere chamber of the microfluidic device was carefully wetted using neurosphere media. After wetting, the neurospheres were transferred into the central neurosphere chamber by the use of a cut pipette tip. After the neurospheres settled to the bottom of the neurosphere chamber the remaining medium was carefully removed employing a gel loading tip. Thereafter, 10 µL of Matrigel^®^ (Corning, 356230) were carefully pipetted into the neurosphere chamber and polymerized for 45 min at 37°C, before adding 50 µL of conditioned neurosphere medium. After 2 days of static culture, a pressure gradient was established in the microfluidic devices by inserting two cut syringes into the media reservoirs on one side of the microfluidic device and adding 600 µL media. Note that as the vascular network developed, the media flow increasingly passed through the low resistance vascular network rather than the gel, causing the pressure to equalize more rapidly, in ∼30 min once fully perfusable. The syringes were refilled every 24 h using fresh VascuLife^®^ medium (LifeLine Technologies, LL-0003) supplemented with 0.19 U/mL of heparin. Neurosphere media was replaced every second day with conditioned media collected from the 96-well plates.

### Supernatant analysis

A meso scale discovery (MSD) assay was employed to analyze the supernatants collected from the 96-well plate. 50 μL were collected from individual wells containing one neurosphere each. The amount of soluble Aβ38, Aβ40, and Aβ42 in the media were measured using the following kits following the manufacturer’s protocol (Meso Scale Discovery: V-plex proinflammatory panel 1 (MSD, K15049D), V-plex chemokine panel 1 (MSD, K15047D), and V-plex Aβ peptide panel 1 6E10 (MSD, K15200E). A volume correction factor was employed to account for the increase in media volume within the 96-well plates over time.

### Permeability assay

Barrier permeability was assessed by sequentially adding 40 µL of a 1 mg/mL fluorescently-labeled 10 kDa or 40 kDa dextran (D1829, Thermo Fisher Scientific) solution in PBS to each of the two media channels of the microfluidic device prior to imaging using a confocal microscope (Olympus, FV-1200). Three regions of interest close to the neurosphere compartment were selected from each device and imaged immediately after the addition of the fluorescently-labeled dextran solution (t = 0) and 9 min (t = 540, 10 kDa) or 12 min later (t = 720, 40 kDa). Images were taken with a resolution of 512 × 512 pixels and a slice dimension of 1,272.32 µm × 1,272.32 µm x 5 µm.

Vascular network permeability was calculated using:

$$\text{Permeability }\left(\text{cm}/s\right)=\frac{R*\left(\frac{1}{t\left(s\right)}\right)*∆I_{M}}{I_{V\left(t=0\right)}-I_{M\left(t=0\right)}}$$

wherein *t* is the time between the two image acquisitions, *I*_*M*_ and *I*_*V*_ are the fluorescence intensity in the matrix and vascular space, respectively and $R=\frac{V_{M}}{SA_{V}}$ where *V*_*M*_ is the matrix volume and SA_V_ is the vascular surface area. The listed parameters were extracted from a macro in ImageJ, developed as previously described (Hajal et al., 2022).

### Aβ42 treatment of vascular networks and ReN-Ctrl co-cultures

Microphysiological systems were treated with 0.001 μg/mL, 1 μg/mL, and 10 μg/mL of Aβ42 (AnaSpec, AS-20276) for 1 week. ReN BBB co-cultures were treated with 1 μg/mL of Aβ42 (AnaSpec, AS-20276) for a duration of 7 days. Pressure gradients were established every 24 h, while the media was replaced every second day. Barrier permeability was assessed at day 7 using 10 kDa dextran.

### Morphological assessment of neurospheres and vascular networks

Both maximum neurite outgrowth [μm] and neurosphere diameter [μm^2^] were determined using the open-source image processing program FIJI (2.9.0/1.53 t). To determine the cross-sectional area of the neurospheres, brightfield images of the neurospheres were converted to 8-bit images; thresholds were adjusted to separate the neurospheres from the background before converting the images to a mask and measuring the area or diameter respectively using the measure function of the program. For the determination of the maximum neurite outgrowth rate, neurites extending from the neurospheres were traced and measured using the freehand lines tool and the measure function, respectively. Morphological parameters of vascular networks were assessed using the open-source image analysis tool “Rapid Editable Analysis of Vessel Elements Routine” (REAVER) and the programming and computing platform MATLAB (R2021b) (Corliss et al., 2020). Stacked images were acquired using a confocal microscope (Olympus, FV-1200) with a resolution of 512 × 512 pixels and a Z-step of 5 µm. Morphological analysis was conducted for three regions of interest per chip, employing maximum intensity projections of images extracted from networks perfused with fluorescently-labeled dextran.

### Immunofluorescence analysis

Prior to the fixation of the microphysiological systems for 24 h using 4% paraformaldehyde at room temperature, microfluidic devices were washed three times by perfusion with PBS. Fixed microphysiological systems were washed three times on a shaker for 5 min each, employing PBS. Subsequently, microvascular networks were permeabilized by perfusion with 0.1% Triton X-100 for 45 min. After washing twice with PBS for 5 min each, the microphysiological systems were blocked at 4°C on a shaker overnight employing a 4% BSA and 0.5% goat serum PBS blocking solution. Following two 5-minute-long consecutive washes at room temperature with PBS, the microfluidic devices were washed again overnight at 4°C on a shaker. Primary antibodies (PDGFRβ (P09619) 1:100, GFAP (P14136) 1:350) were diluted in PBS and incubated overnight at 4°C on a shaker. After two 5-minute-long consecutive washes at room temperature with PBS and an overnight wash at 4°C, PBS was removed and a 1:200 dilution of secondary antibodies and a 1:1,000 dilution of the nuclear dye DAPI was applied to the microphysiological systems. After an overnight incubation step at 4°C the microfluidic devices were washed three times at room temperature before a final overnight wash at 4°C employing a shaking platform. Images were acquired using a confocal microscope (Olympus, FV-1200).

For the Aβ staining, the microfluidic devices were blocked overnight. The next day the devices were washed twice with PBS. After permeabilization with 1X TBST (Boston BioProducts—IBB-580X) containing 0.5% Triton X-100 (Millipore Sigma, X-100) and 4% donkey serum (abcam, ab7475), the 3D6 antibody (a gift from Lilly, 1:500) was added and incubated overnight. The next day, the devices were washed three times with PBS. This is followed by incubating with the secondary antibody, Cy5 Rabbit (Jackson, 711-175-152, 1:400) overnight. The following day the devices were washed twice with PBS and, once overnight. Microfluidic devices were then imaged using a Nikon A1 HD25 confocal microscope, and images were processed using ImageJ2 (Version 2.3.0).

### Statistical analysis

Statistical analysis and data visualization was conducted using the biostatistics program GraphPad Prism 9. Welch’s t-tests, Mann-Whitney tests, one-way ANOVAs, or two-way ANOVAs combined with Tukey’s multiple comparison tests were performed to assess statistical significance. Normality was tested using a combination of the Shapiro-Wilk test and the Kolmogorov-Smirnov test.

## Results

### Microfluidic co-culture enables the generation of perfusable microvascular networks in contact with neurospheres

**FIGURE 2:**
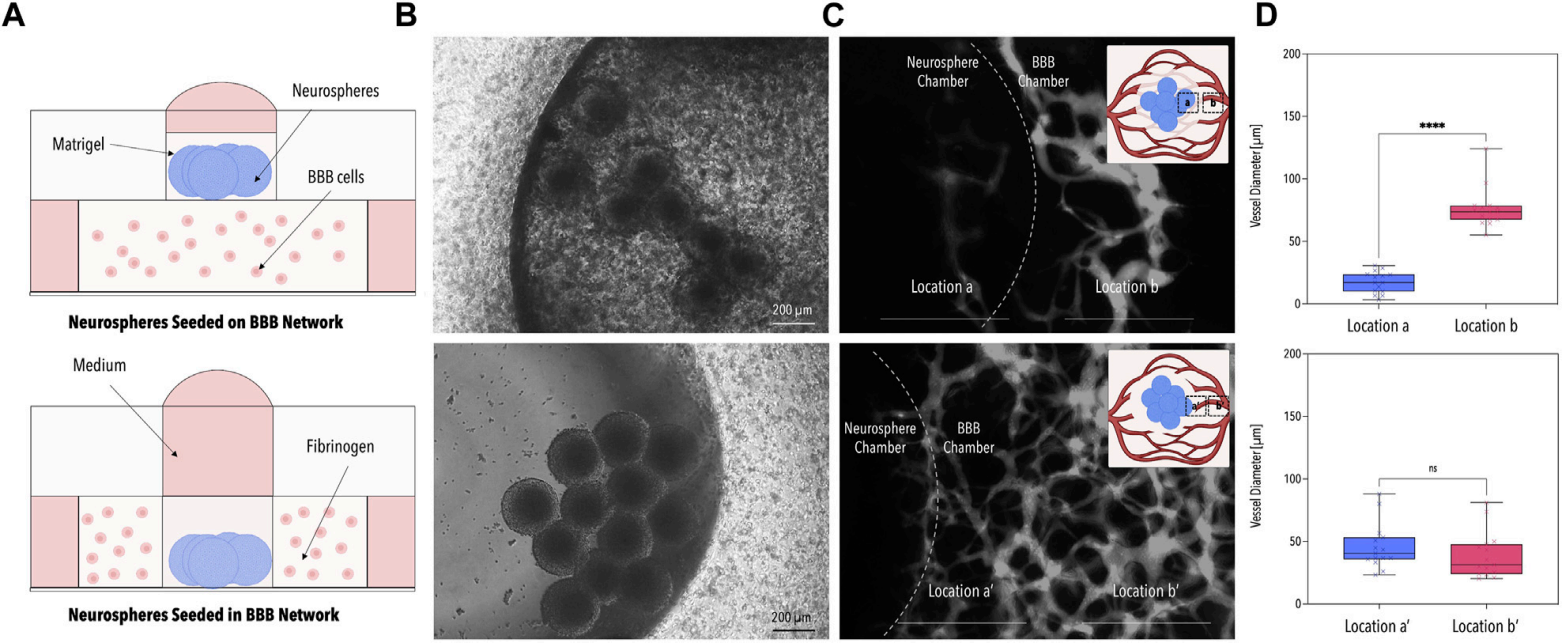
Optimization of the Microfluidic Co-Culture Method. **(A)** Schematic illustrations of the two tested methods of co-culture. Neurospheres were seeded either on the BBB network (top panel) or within the BBB network (bottom panel). **(B)** Phase contrast images of the neurospheres for the two co-culture methods. **(C)** Fluorescence images of the 40 kDa fluorescently-labeled dextran perfused through the BBB network. The dotted line indicates the border of the neurosphere chamber. **(D)** Average BBB vessel diameter at locations a, b, a’, and b’ of image **(C)** [*n* = 3, *p* < 0.001 (****)]. Created with BioRender.com.

To account for the differing requirements of the individual cells of the neurovascular unit and to enable direct cell-to-cell interactions a microfluidic device was developed and tested as part of this study. As depicted in Figure 1 the PDMS-based microfluidic platform is comprised of three interconnected channels: one central BBB chamber and two parallelly aligned media channels. A cylindrical recess further connects the central BBB chamber to an additional media reservoir on top of the microfluidic co-culture platform. A multi-step seeding protocol is developed encompassing 1) the loading of the entire central chamber with fibrinogen-embedded primary cells of the human BBB through the loading port BBB (indicated in Figure 1C), 2) the optional removal of a defined volume of fibrin to empty the cylindrical recess and 3) the injection of Matrigel^®^-encapsulated neuronal microtissues via the top access port (indicated in Figure 1C as neurosphere compartment). Neuronal cells can either be embedded within or on top of a self-assembling vascular network of the BBB depending on whether step (2) is included. At the same time, differing media requirements are taken into account through the use of three separate media channels. While the formation of the vascular network is supported by the application of a pressure gradient from one side of the media channels to the other, nutrient supply to the neuronal compartment is ensured by the cylindrical reservoir located on top of the PDMS-based device.

In the first series of experiments, the optimal co-culture set-up was identified by comparing two different seeding strategies: (i) embedding ReN-Ctrl neurospheres on top of the capillary bed (Figures 2A, B top panel) and (ii) embedding ReN-Ctrl neuronal microtissues within the central BBB chamber (Figures 2A, B bottom panel). While both strategies resulted in the formation of vascular networks, marked differences in network perfusability and morphology were observed. Embedding neuronal microtissues on top of the capillary bed resulted in distinctly lower network perfusion, caused by a significant reduction in vessel diameters (to ∼10 µm) beneath the neuronal microtissues, characteristic of vascular regression (Figures 2C, D top panel). In contrast, introducing neurospheres into the central BBB chamber resulted in perfusable networks including open lumens within the immediate surroundings of the ReN-Ctrl neurospheres (Figure 2C bottom panel). Furthermore, no significant differences in vessel diameters were observed when comparing capillaries at the border of the microfluidic device with vessels close to the center of the platform or close to the neurospheres respectively (Figure 2D bottom panel). To that end, the second approach was selected for all further experiments.

### Pre-differentiated ReN-AD neurospheres display AD-specific phenotypes

**FIGURE 3:**
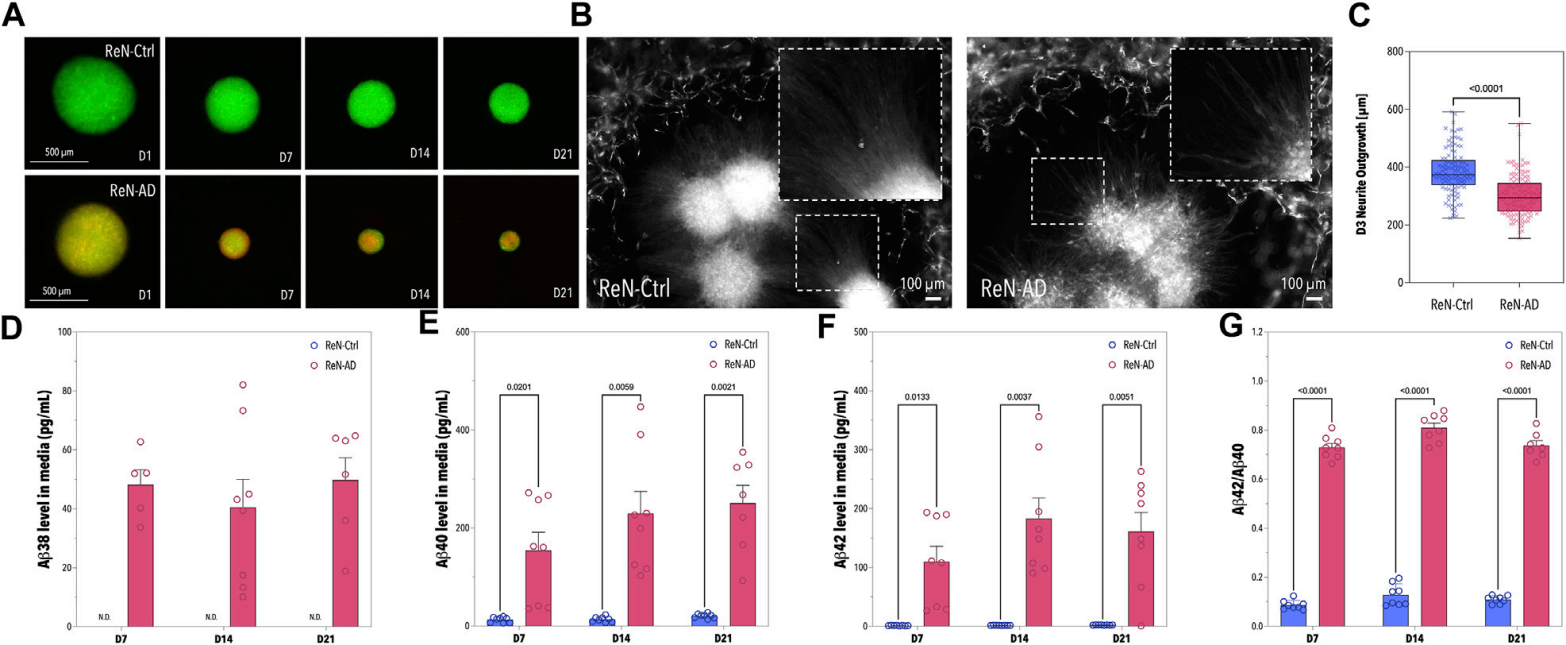
Pre-differentiation of ReN-Ctrl and ReN-AD Neurospheres. **(A)** Fluorescence images of neurospheres formed with ReN-Ctrl cells (tagged with GFP) and ReN-AD cells (tagged with GFP and mCherry) obtained on day 1, 7, 14, and 21 of differentiation. **(B)** Fluorescence images of the ReN-Ctrl and ReN-AD neurospheres seeded within the co-culture microfluidic devices. The images were obtained on day 3. **(C)** Box-plot depicting the neurite outgrowth of ReN-Ctrl and ReN-AD neurospheres on day 3 of the microfluidic co-culture (*n* ≥ 5, 2 independent experiments). **(D–G)** Concentrations of soluble Aβ38, Aβ40, and Aβ42 in the supernatant of ReN-Ctrl and ReN-AD neurospheres, as well as the corresponding Aβ42/Aβ40 ratio on day 7, 14, and 21 of differentiation. Data obtained by MSD analysis (*n* = 3, 3 independent experiments). Created with BioRender.com.

To demonstrate the ability of ReN-AD microtissues to emulate disease-specific phenotypes *in vitro*, ReN-Ctrl and ReN-AD cell-derived neurospheres were cultivated for 21 days while time-dependent changes in morphology and Aβ secretion were assessed. Overall, distinct differences in microtissue size between ReN-Ctrl (GFP-tagged) and ReN-AD (GFP- and mCherry-tagged) neurospheres were observed over the cultivation period (Figure 3A). In both groups, neuronal microtissues decreased significantly in size over time. Diameters of the ReN-Ctrl neurospheres decreased from initial values on day 1 by 35% on day 7, 42% on day 14, and 50% on day 21, whereas diameters of the ReN-AD neurospheres decreased by 50% on day 7, 62% on day 14, and 75% on day 21.

To confirm the presence of soluble Aβ38, Aβ40, and Aβ42 in the supernatant of our ReN-AD neurosphere model, cell culture media was collected on day 7, 14, and 21 of differentiation (Figures 3D–G). In line with our previous studies, Aβ38, Aβ40, Aβ42 levels, and Aβ42/40 ratios in the medium were significantly greater for ReN-AD neurospheres compared to ReN-Ctrl neurospheres in the course of the 21-day differentiation period (Figure 3D–G). In addition, while Aβ40 levels significantly increased over time from 154.13 ± 105.53 pg/mL on day 7–250.65 ± 96.14 pg/mL on day 21 (*p* = 0.0038), soluble Aβ42 levels slightly decreased from 183.19 ± 98.50 pg/mL on day 14–161.14 ± 91.38 pg/mL on day 21, likely due to an aggregation of Aβ42 within the 3D ReN-AD cell cultures. This observation is further supported by a slight decrease in the Aβ42/40 ratio from 0.809 ± 0.056 at day 14 to 0.737 ± 0.052 at day 21.

### Microfluidic model replicates AD-specific phenotypes *in Vitro*

**FIGURE 4:**
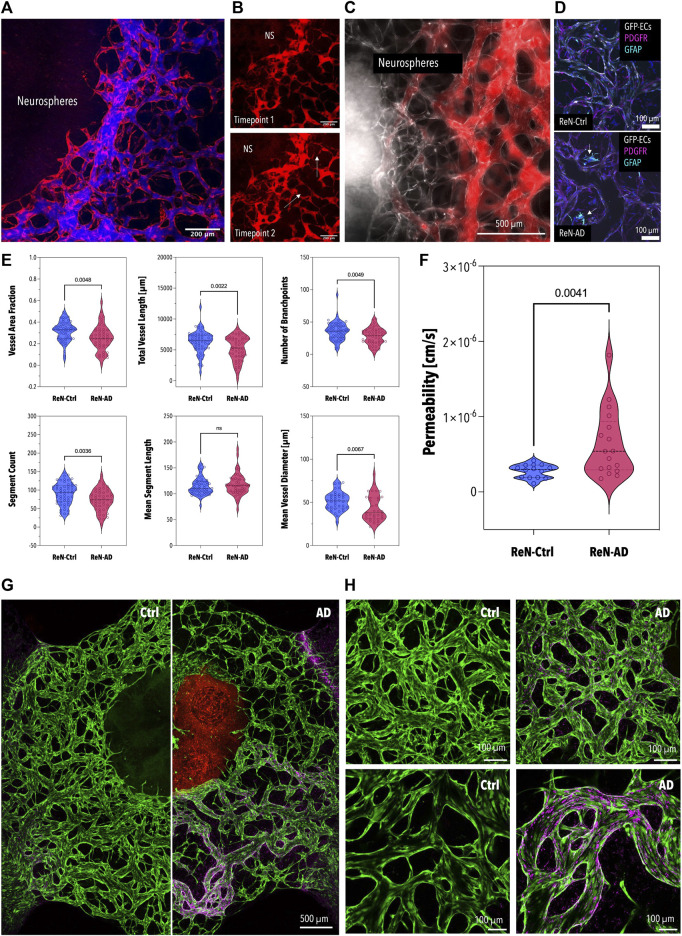
Permeability and Morphology of the BBB Microvascular Network Co-Cultured with the Neurospheres and Aβ Deposition in Microvascular Networks in Co-Culture with ReN-Ctrl and ReN-AD Neurospheres **(A)** Fluorescence image of the BBB network (red) perfused with 40 kDa fluorescently-labeled dextran (blue). **(B)** Fluorescence image of a self-assembled network of the human BBB in direct contact with ReN-Ctrl neurospheres (GFP; gray) after 7 days of co-culture. The network was perfused with a 40 kDa fluorescently-labeled dextran (red). **(C)** Immunofluorescence images of vascular networks after 7 days of co-culture with ReN-Ctrl (top panel) and ReN-AD (bottom panel) neurospheres. Endothelial cells (GFP) are labeled in grey, pericytes (PDGFR) are labeled in magenta, and astrocytes (GFAP) are labeled in cyan. Arrows indicate activated astrocytes in ReN-AD co-cultures. **(D)** Comparative analysis of morphological parameters extracted from BBB networks after 7 days of co-culture with ReN-Ctrl and ReN-AD neurospheres (*n* ≥ 4, 3 independent experiments). **(E)** Left panel: violin plot depicting the permeability values calculated for BBB networks after 7 days of co-culture with ReN-Ctrl neurospheres and ReN-AD neurospheres (*n* ≥ 4, 3 independent experiments). Right panel: perfused vascular network at timepoint 1 (right after perfusion; top panel) and timepoint 2 (12 min later; bottom panel). ReN-AD neurospheres are indicated as NS; arrows indicate dextran leakage. Overview **(F)** and representative **(G)** immunofluorescence images depicting spatially localized 3D6-positive Aβ deposition (Cy5) in microvascular networks of the BBB (GFP) after 7 days of co-culture with ReN-Ctrl and ReN-AD neurospheres. Created with BioRender.com.

Finally, to assess the ability of our model to emulate AD-specific phenotypes *in vitro* neurospheres were introduced into the microfluidic platform on day 21 of differentiation and co-cultivated with microvascular cells for 7 days. In addition to the significant differences in microtissue size, reduced neuronal outgrowth, an indicator of neuronal health, was observed in pre-differentiated ReN-AD neuronal microtissues after 3 days of co-culture (Figures 3B, C). While both co-cultures resulted in perfusable vascular networks close to the neuronal microtissues (Figures 4A, B), a comparative analysis of vascular networks revealed distinct morphological differences between ReN-Ctrl and ReN-AD vascular networks (Figures 4C, D). In particular, microvascular networks co-cultured with ReN-AD neurospheres displayed a 1.23-fold reduction in average vessel area fraction, a 1.22-fold decrease in total vascular network length, a 1.27-fold reduction in branch points and a 1.23-fold reduction in segment counts as well as a 1.19-fold reduction in vessel diameter, pointing towards reduced vessel complexity under pathological co-culture conditions (Figure 4D). Furthermore, co-culture resulted in a significant increase in vascular permeability from 2.8 × 10^−7^ cm/s for ReN-Ctrl cultures to 6.4 × 10^−7^ cm/s for ReN-AD cultures, highlighting the ability of the microfluidic model to capture critical pathological phenotypes of the disease *in vitro* (Figure 4E).

To confirm whether the increase in vascular permeability can be linked to secreted Aβ, vascular networks were treated with increasing concentrations of synthetic Aβ42 (Supplementary Figure S1). As depicted in Supplementary Figure 1B impaired barrier integrity was observed with increasing concentrations of Aβ42 (0.001 µg/mL to 10 μg/mL). While the control networks displayed a permeability value of 3.6 × 10^−7^ cm/s, treatment with 10 µg/mL of Aβ42 resulted in a permeability of 5.3 × 10^−7^ cm/s (10 kDa dextran) (Supplementary Figure 1B left panel). In addition to impaired barrier permeability the networks displayed a significant reduction in perfusable vessels (Supplementary Figure 1A), with the volume fraction of perfusable networks decreasing from 0.37 to 0.20 (Supplementary Figure 1B right panel). Highlighting the importance of paracrine signaling in Aβ-mediated vascular dysfunction, a significant increase in barrier permeability (2-fold) was observed upon treating microvascular networks in co-culture with ReN-Ctrl neurospheres with 1 µg/mL of Aβ42 (Supplementary Figure 1C).

Lastly, plaque formation within microphysiological systems was probed by staining vascular networks with a 3D6 antibody. While no statistically significant differences were observed between ReN-Ctrl and ReN-AD networks, clear localized Aβ deposition was observed in the vascular networks co-cultured with ReN-AD as compared to ReN-Ctrl neurospheres (Figures 4F, G; Supplementary Figure S2). The heterogeneous distribution of Aβ plaques in the vascular networks potentially corroborates the high variability observed in the permeability of ReN-AD co-cultures (Figure 4E).

## Discussion

In this study, we present a novel AD model combining a 3D self-assembled BBB vascular network with neuronal microtissues within one system. Specific focus was directed toward addressing long culture periods required for neuronal tissue differentiation and differing cellular requirements between the individual constituents of the neurovascular unit. This was accomplished by pre-differentiating neuronal microtissues to express AD-specific phenotypes prior to co-culture in a multi-compartmentalized microfluidic device.

The presented model is superior to our previously reported AD model as it combines self-assembled networks of the human BBB comprising brain microvascular endothelial cells, astrocytes, and brain pericytes with AD-neurons, as opposed to brain microvascular endothelial cells and neurons only (Shin et al., 2019). To reduce co-culture times on chip ReN-AD neurospheres were pre-differentiated for a period of 21 days. Next to reduced microtissue size, the ReN-AD neurospheres displayed disease-specific phenotypes including a significant increase in soluble Aβ40 levels. Reduced engineered neuronal tissue size has previously been reported and was linked to pathological alterations including both genetic changes and altered rates of neuronal proliferation or differentiation (Toyoshima et al., 2016; Amin and Paşca, 2018; Xu and Wen, 2021). This observation is further supported by a significant reduction in neuronal outgrowth detected for ReN-AD neurospheres, indicative of impaired neuronal health (Su et al., 1998; Blazquez-Llorca et al., 2017). In addition, dystrophic neurites, commonly observed in AD, were detected extending from ReN-AD neurospheres (Figure 3B).

Initial assessment of the microfluidic co-culture platform revealed better outcomes including a homogenous vessel diameter distribution and fully perfusable vasculature when placing neuronal microtissues beside the vascular network of the BBB compared to seeding neurospheres on top of the capillary bed (Figures 2A, B). In the second approach, the interface between the two hydrogel matrices is doubled, thus potentially increasing the negative effect of the neurosphere media on the vasculature.

A co-cultivation period of 7 days was sufficient to significantly alter both vascular morphology and integrity in the case of Aβ-secreting neuronal microtissues. In agreement with post-mortem studies on AD patients, capillary networks in co-culture with ReN-AD neurospheres displayed a significant reduction in both vessel length and density as well as in vessel diameters (Brown and Thore, 2010; Steinman et al., 2021). Furthermore, microfluidic co-culture demonstrated impaired barrier integrity, as indicated by a significant increase in vascular permeability to a 40 kDa fluorescently-labeled dextran. This observation aligns with previous studies linking Aβ to oxidative stress-mediated vascular dysfunction. In contrast, the permeability of the BBB network co-cultured with ReN-Ctrl neurospheres was comparable to *in vivo* models (Yuan et al., 2009; Shi et al., 2014).

Lastly, Aβ deposition was observed in some regions of the microvascular networks in co-culture with ReN-AD neurospheres, pointing towards the recapitulation of amyloid angiopathy-associated phenotypes within the co-culture model.

While our system recapitulates AD-associated phenotypes *in vitro* by interconnecting AD-neurospheres with a microvascular network of the BBB, further investigation is clearly warranted. To illustrate, during the 7 days of co-culture, the vascular medium was replenished every day, leading to a depletion of secreted factors in all compartments of the model as well as a transient flow through the vascular network. Furthermore, as the vascular networks develop and become increasingly perfusable, the duration of transient flow progressively decreases. We, therefore, suggest that continuously re-circulating the medium through the microvascular network might enhance potential pathological alterations by supporting the distribution of secreted factors within the brain interstitial space and the intravascular compartment. In addition, physiological levels of flow would likely cause a reduction in permeability as demonstrated in other studies (Offeddu et al., 2021; Haase et al., 2022). The duration of the microfluidic co-culture may be extended to observe time-dependent pathological phenotypes, including pronounced Aβ plaque formation, which has been proposed to follow BBB dysfunction *in vivo* (Paul et al., 2007; Sagare et al., 2013; Montagne et al., 2017; Sweeney et al., 2018). In addition, future studies may further investigate the interrelationship between vascular permeability and spatially localized Aβ deposition. In summary, we believe that our system holds significant value in examining the role of the BBB in AD. Furthermore, the methodology can be seamlessly expanded to integrate patient-specific brain organoids or neurospheres in the future, enabling the study of neurovascular-associated pathological phenotypes in as little as 7 days.

## Conclusion

We have successfully demonstrated a method for generating a microphysiological AD model that recapitulates critical aspects of the pathology *in vivo*. Our system offers reduced co-cultivation times, while still enabling the observation of pathological changes. Overall, the presented approach holds great potential for studying the role of the BBB in the onset and progression of various neurodegenerative diseases (e.g., AD, Parkinson’s or Huntington’s Disease) that, moreover, can be employed for drug screening studies.

## Data Availability

The original contributions presented in the study are included in the article/Supplementary Material, further inquiries can be directed to the corresponding author.
