# Supplementary material for: Accelerating the in vitro emulation of Alzheimer’s disease-associated phenotypes using a novel 3D blood-brain barrier neurosphere co-culture model
Source: Front Bioeng Biotechnol. 2023 Oct 9;11:1251195. doi: 10.3389/fbioe.2023.1251195 (PMC10600382; doi:10.3389/fbioe.2023.1251195)
Supplement: Supplementary file 1 [file DataSheet1.docx]

**Supporting Information**


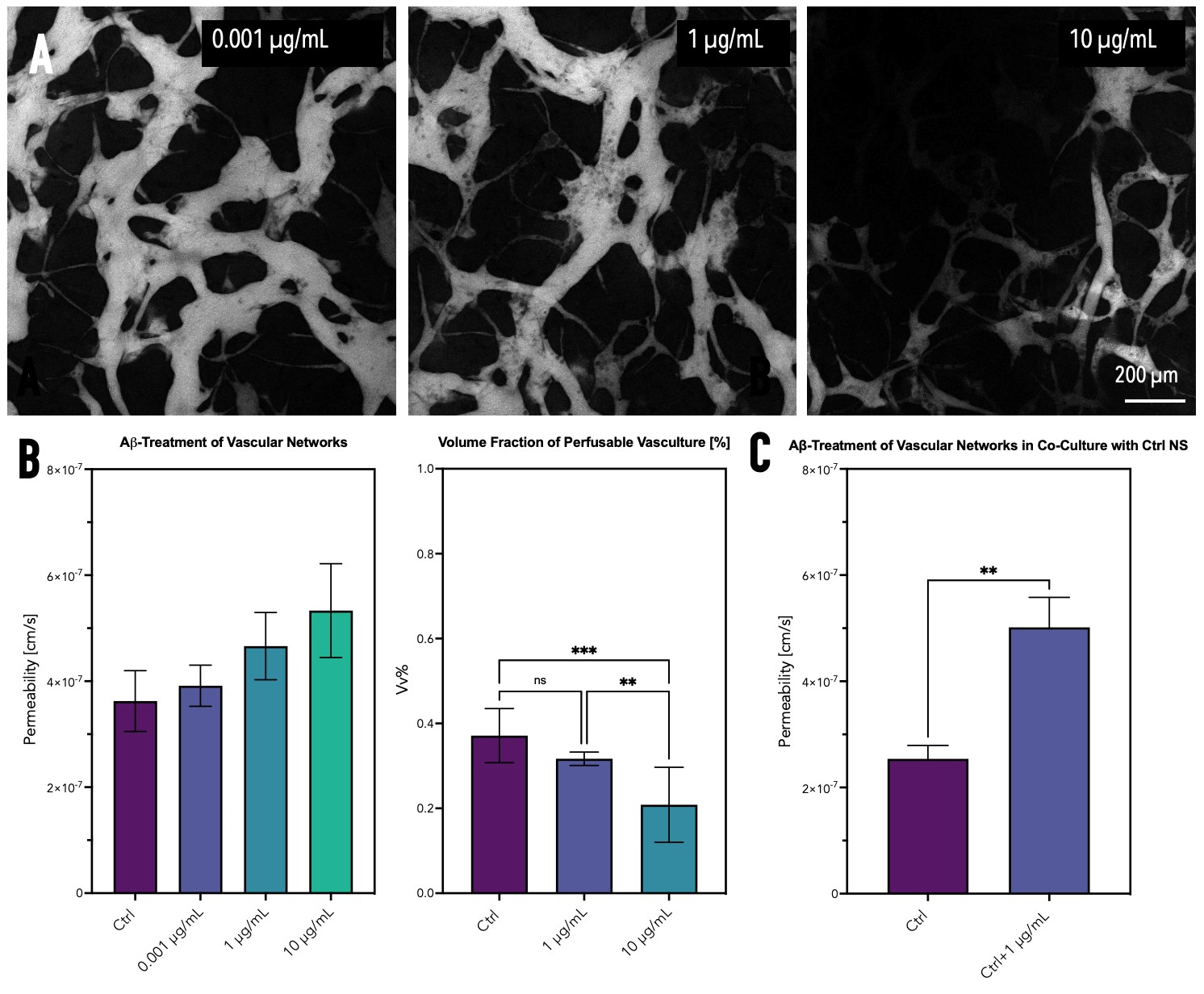


**Figure SI 1. Aβ42 Treatment in Microvascular Networks of the BBB**

(A) Representative immunofluorescence images of perfused (10 kDa fluorescently-labeled dextran) vascular networks treated with 0.001 µg/mL, 1 µg/mL, and 10 µg/mL of Aβ42. (B) Permeability (left panel) and perfusable vessel fraction (right panel) analysis for vascular networks after 7 days of Aβ42 treatment. (C) Permeability analysis for ReN-Ctrl co-cultures treated with 1 µg/ml Aβ42. (n ≥ 3, 1 to 2 independent experiments).


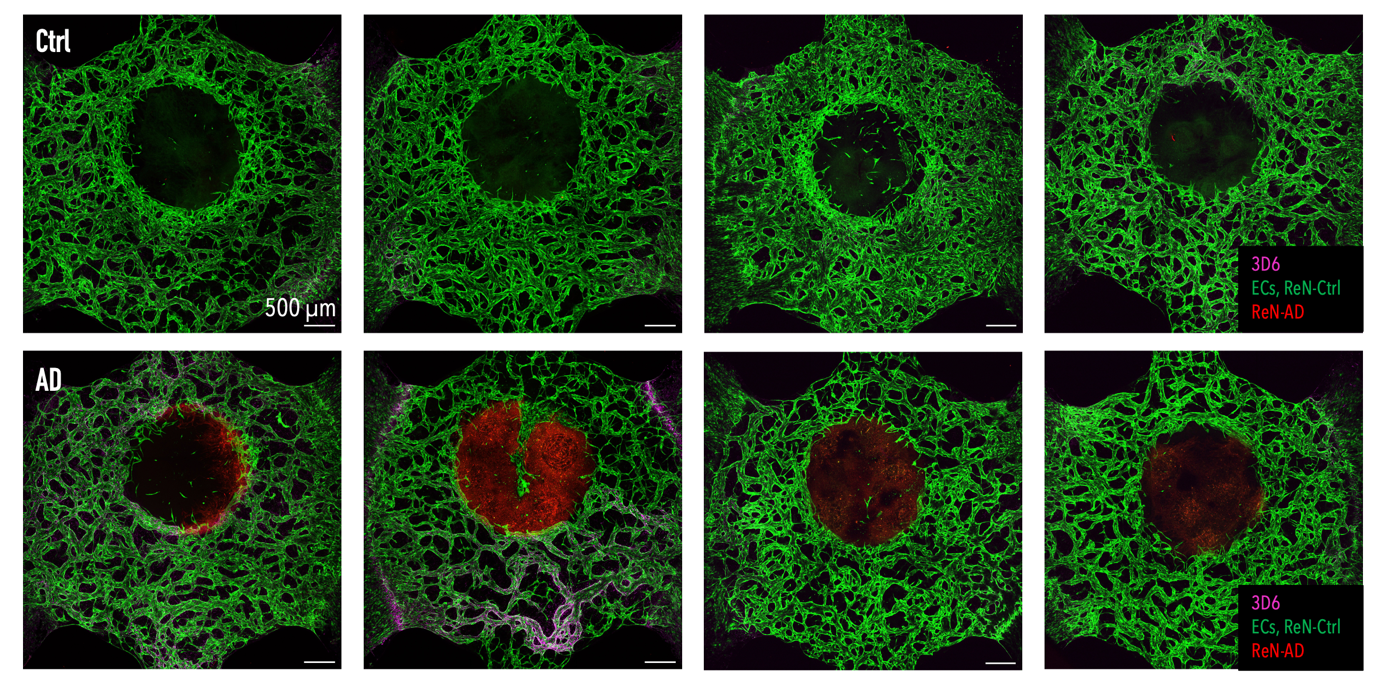


**Figure SI 2. Aβ Deposition in Microvascular Networks**

Overview immunofluorescence images depicting spatially localized 3D6-positive Aβ deposition (Cy5) in individual microvascular networks of the BBB (GFP) after 7 days of co-culture with ReN-Ctrl and ReN-AD neurospheres. The top panel displays microvascular networks of the BBB (GFP) co-cultured with ReN-Ctrl neurospheres (GFP); the bottom panel shows microvascular networks of the BBB (GFP) co-cultured with ReN-AD neurospheres (mCherry).
